# Distributed Ledger Technology for eHealth Identity Privacy: State of The Art and Future Perspective

**DOI:** 10.3390/s20020483

**Published:** 2020-01-15

**Authors:** Mohammed Amine Bouras, Qinghua Lu, Fan Zhang, Yueliang Wan, Tao Zhang, Huansheng Ning

**Affiliations:** 1 The School of Computer and Communication Engineering, University of Science and Technology, Beijing 100083, China; bouras.ma@xs.ustb.edu.cn (M.A.B.); fanzhang@xs.ustb.edu.cn (F.Z.); 2 The Commonwealth Scientific and Industrial Research Organisation (CSIRO) Data 61, Eveleigh NSW 2015, Australia; qinghua.Lu@data61.csiro.au; 3 Run Technologies Company, Ltd., Beijing 100192, China; yueliang@bjrun.com; 4 Beijing Engineering Research Center for Cyberspace Data Analysis and Applications, Beijing 100083, China; 5 Key Lab of Information Network Security, Ministry of Public Security, Shanghai 200031, China

**Keywords:** blockchain, identity management, eHealth, distributed ledger technology, digital identity

## Abstract

Electronic healthcare (eHealth) identity management (IdM) is a pivotal feature in the eHealth system. Distributed ledger technology (DLT) is an emerging technology that can achieve agreements of transactional data states in a decentralized way. Building identity management systems using Blockchain can enable patients to fully control their own identity and provide increased confidence in data immutability and availability. This paper presents the state of the art of decentralized identity management using Blockchain and highlights the possible opportunities for adopting the decentralized identity management approaches for future health identity systems. First, we summarize eHealth identity management scenarios. Furthermore, we investigate the existing decentralized identity management solutions and present decentralized identity models. In addition, we discuss the current decentralized identity projects and identify new challenges based on the existing solutions and the limitations when applying it to healthcare as a particular use case.

## 1. Introduction

Electronic health (eHealth) refers to the use of information and communication technologies to improve the quality of healthcare. The aim of eHealth is involving multiple clinics, different care units and departments to make the healthcare systems management more efficient, and deliver convenient care services with good patient experience [1]. Further, giving a timely health care record to a patient after collecting the physical data from different resources and run a diagnosis by physicians or third-party service providers is the main goal of eHealth which is making the medical data available anytime, anywhere. The healthcare system is extremely mobile in nature and working in such systems requires a high mobility of physicians and caregivers as well as collaboration among different decision-makers and stockholders including care professionals and patients [2]. eHealth identity management is much more than just authorizing or revoking access to certain entities; it is the process of protecting sensitive data including authentications of users, managing the medical records, and guarantees the security and privacy of entities according to the local legislation and laws [3].

The traditional identity management system (IDM), also known as identity and access management (IAM), adopted the centralized approach, and such design already witnessed several limitations and weaknesses regarding security, privacy, and scalability. The negative side of centralized models is that identity providers have full control over individuals’ identity, and the identity owners are incapable of preventing any misuse of their identities. Moreover, centralized databases are attractive for hackers, and breach incidents are increasing every day due to weak security which leads to identity theft and sensitive data disclosure. Likewise, identity owners are always required to share their credentials in order to register or access to a service without standard or regulation on what data have to share as well as it gets stored in a different database across the internet which it leads to oversharing data that can be used by hackers as well.

Distributed ledger technology (DLT) is an umbrella term used to describe the technological infrastructure that allows records and information to be distributed in an immutable ledger across the network of those using it. The term is being used to unify the different impacts of the blockchain, hybrid blockchain, directed acyclic graph, and other decentralized protocols on diverse domains and sectors [4]. DLT identity management is a promising approach to solve the existing digital identity limitation, with the potential of ensuring integrity while sharing information between different healthcare stockholders and grant access to their network without compromising data. Self-sovereign identity (SSI) model is the next identity model that will be implemented on the top of DLT due to its perspective of moving the identity access and management toward users, and make individuals independent from any identity providers [5].

This paper’s contribution is to give a comprehensive review and state of the art of the existing centralized identity management and the advancement of decentralized identity management models, as well as discussing the eHealth identity management (IdM) roles, key players, tasks, and identity scenarios. Together with, highlighting the limitation of centralized identity models and presenting the opportunities of using DLT for eHealth decentralized identity management. Different from other works, we enumerate and classify different healthcare identity scenarios and use cases to underline the requirement of such systems. Identically, we present all the existing solutions that support DLT, self-sovereign identity, and decentralized identity models by discussing the potential of implementing such solutions for eHealth, and we deliberate the benchmarks of evaluating blockchain-based identity management solutions. Accordingly, we elaborate on distinct research challenges and future works.

The rest of the paper is organized as follows. In Section 2, we present the background and related works of identity management and distributed ledger technologies. Section 3, we quote the evolution of identity management models together with a detailed comparison. Section 4, we identify the key players of eHealth systems and the role of each player according to healthcare identity management scenarios. In Section 5 and Section 6, we present the existing decentralized identity models together with the existing identity management solutions implemented on the top of DLTs. Finally, we settle the work by identifying different challenges and future works, alongside with a conclusion.

## 2. Background and Related Work

### 2.1. Identity and Identity Management

An identity is a set of credentials and identifiers of entities represented in a particular context such as entity name, entity ID, and different entity attributes [6,7]. Likewise, a digital identity is the representation of an entity attributes in a digital way in order to access the systems and applications using the identity mediation process [8]. In general, the digital identity is divided into nyms and partial identity, where nyms identity is to give a user an anonymous identity, yet partial identity is to associate a set of real attributes to an individual [9]. Similarly, other scholars categorize identity as a strong and weak identity [10]. Identity management is the process and the framework used to control and manage identities in cyber and cyber-physical systems [11]. IdM is an integrated system that includes authentication of entities, authorization and access privileges methods, and the whole lifecycle process of digital identity like creation, updating, and maintenance by following a set of rules and conditions provided by the moderator of the identity system. In general, the core system of IdM includes three main entities which are a user, identity provider (IdP), and service provider (SP) [12].

Many scholars and researchers had classified the existing identity management approaches and methods to three models which are isolated model, centralized model, and federated model [12]. Moreover, Mullyniemi [13] gives a grouping of federated IdM, small-scale IdM, and proprietary IdM based on the intended domain and magnitude of the existing IdM solutions. In like manner, three IdM paradigms were proposed by Cao et al. [12] including network-centric, service-centric and user-centric paradigms.

### 2.2. Distributed Ledger Technology

Distributed ledger technology is an innovative method of storing records, transactions, contracts and agreements within and between organizations [14]. A distributed digital ledger is a distributed database run by nodes in a peer to peer network and preserved by consensus algorithms, different from a centralized approach where the consensus protocols play the role of the central administration. DLT is different from the centralized network where the information is distributed in a network of nodes and for any update or change in the network, the operation will be echoed immediately for all holders of the ledger, at the same time multiple copies are stored in different machines [15].

The blockchain is the first DLT technological infrastructure, originally developed to support Bitcoin crypto-currency, a type of immutable ledger that group a set of records in a block, and each block is chained to the succeeding block using a cryptographic signature. The three types of blockchain are public, private and federated blockchains [16]. A public blockchain is based on open-source consensus protocols to achieve reliability in a network involving multiple nodes such as proof of work (PoW), practical Byzantine fault tolerance (PBFT), proof of stake (PoS). Anyone in the world can participate, send or read the transactions as well as validating transactions after running a public node in their local device [17]. In the Private blockchain, annotating the rules are given to a centralized organization, only participants with permission can execute operations on the chained ledger [18]. Federated blockchain or consortium blockchain runs under a group of trusted organizations, and each organization operates one or more nodes, and a group of selected nodes control the consensus process of the federated chained ledger. Federated blockchain is faster, provides more privacy on transactions, with higher scalability [19].

Furthermore, DLT can be classified regarding the types of ledgers to permissionless and permissioned ledgers. The permissionless ledger allows anyone in the network to contribute data and be part of the consensus process and everyone has a copy of the ledger. In other words, the ledgers cannot be owned by a particular organization, yet no one can prevent a transaction from being injected to the ledger, and all the nodes participate to maintain the integrity of the ledger [20]. On the contrary, permissioned ledgers can be owned by one or more organizations, different from the permissionless concept, where the consensus process is simple and faster because the integrity of the ledger is checked by a set of trusted nodes. As a result of limiting the number of trusted nodes, the consensus process provides a digital signature between the owners and gives the ledger a high degree of confirmable data and records security [15].

### 2.3. Blockchain-Based Identity Management Systems

DLT is rapidly transforming the digital world, and the potential of blockchain to the other domains is heavily investigated by the research community. Many works have already exploited blockchain-based identity management systems. First, Kshetri mention and explained in the article [21] that blockchain-based identity management can strengthen the IoT security and trust between the devices and users as blockchain already used to securely store important data like digital rights and identity credentials. Moreover, Lin et al. [22] designed a new authentication scheme for blockchain-based identity management that uses the immutability of nodes as a feature to leverage strong transitively closed undirected graph authentication (TCUGA) scheme. Similarly, Lee [23] used blockchain to introduce the concept of ID as a service (IDaaS) for identity and authentication management systems. Jamila et al. [24] proposed Blockchain-based identity management to secure personal data sharing over the networks, and they illuminated the need for the blockchain and decentralized self-sovereign identities. Given those research points, mainly, the authors are trying to use blockchain and its features as the backbone of identity management regardless of the domain of use. On the other hand, healthcare identity management is a domain where the IT is hardly adopted as the most solutions are not designed for healthcare.

Moreover, Yun Lim et al. [25] gave a summary of blockchain-based identity management and authentication solutions for the last five years together with identifying centralized identity management open issues and challenges. Similarly, Zhu et al. [26] investigated recent surging blockchain-based identity management for the internet of things (IoT) and enumerate several identity projects and startups in the era of IoT. Different from the previous research works, our paper aims to provide an overview of the existing decentralized solutions that may be of good use to the eHealth identity management by enumerating current DLT-based identity solutions that are already in development and production stages. Also to provide a deep understanding of decentralized identity management systems and its features while giving an extensive understanding of roles, tasks, and scenarios of healthcare applications.

With the growth of distributed ledger technology and its huge impact on identity management, the classification of IdM has been upgraded by adding a new model which is the decentralized identity model that has been omitted in all previous works. The contributions of this paper are as follow:

- A comprehensive review of the identity management models evolution.
- Enumerating the limitation of the centralized identity toward eHealth.
- Identification of healthcare key players, roles, and tasks, as well as eHealth identity scenarios.
- A number of challenges and future works are also discussed.

## 3. The Evolution of Identity Management Models

### 3.1. The Evolution and the Stages of Identity Models

#### 3.1.1. Centralized Identity Model

A centralized identity is implemented in a client/server model, and it separates the service provider (SP) and identity provider (IDP) functions. IdP server contains the user identity and user authentication functions that is used by all services, besides SPs avoid storing identities locally, all the requests are sent to the centralized IdP server to authenticate users. [12]. The centralized model is suitable for managing a lot of users, and it can be implemented in many different ways namely single sign-on [27], identifier domain, and Meta-identifier domain [28]. Several identity management systems are materialized using the centralized model notably PKI [29], and Kerberos [30]. However, storing all the identities in a single IdP is an ineffective way to support user privilege delegation and cross-domain access, while also this model shows weak privacy protection [28].

#### 3.1.2. Federated Identity Model

This model proposed to tackle the challenge of cross-domain access by integrating different domains together and make it a unique global domain virtually [31]. Many scholars defined the federated model as the set of agreements, standards, and technologies that enable a group of SPs to recognize and approve user identifiers from other SPs within a federated trust group [32]. The idea behind the federated approach is to link different user identifiers across multiple SPs, and allow the user to authenticate with the same credential among those SPs, and also be considered identified by all the SPs as well. In fact, each SP store users’ identities locally and provide a separate identifier and credential to the same user, but the user likely will not use them all and the access will be provided through a single SP. Several protocols and IdM systems implemented federated model including security assertion markup language (SAML) [33], Liberty Alliance framework [34] and Shibboleth [35] open source project of the federated model. However, this model has several challenges like misusage of identities and the lack of an effective mechanism to keep uniformity and correctness of user information [28].

#### 3.1.3. User-Centric Identity Model

User-centric model is proposed to tackle the limitations of the models described above, by the need to facilitate the user experience. A user is inapt to memorize the growing number of passwords and credentials as there are many federated domains with different levels of sensitivity that require a different type of credentials. The idea behind the user-centric model is to provide a mechanism that let users store different identifiers and credentials in one place, and give the user complete control of their digital identity with the ability to share an identity from a service to another. User-centric identity can be combined with any identity model regardless of the way of authentication and access as the user must authenticate himself to the personal authentication device (PAD) first, and the PAD will be used for authentication to any service provider [31]. This model creates a better user experience with much more control over a user identity which is adopted by big firms like Google and Facebook under the name of identity 2.0, yet the model failed his mission as still the user is not the owner of his identity and if the firm deletes his account he won’t be able to use it again.

#### 3.1.4. Decentralized Identity

The current identity challenges tie back to the concept of centralized design, as more data we collect and store, security and privacy concerns appear. The aims of the decentralized identity are to hand back the responsibility to the users and give them the power and ability to control and manage their own identity. The decentralized identity can be achieved by using the emerging distributed ledger technology as it provides a decentralized and secure way to store data where no organization control or have the ability to change the network, and the trust is reached by consensus mechanism between network nodes. The decentralized identity is still in early-stage development and it needs more discussion and investigation. The decentralized community is divided between two decentralized implementation models which are the self-serving identity and decentralized trusted identities. The models are discussed in the decentralized identity management models section.

### 3.2. Comparison of Identity Management Models

#### 3.2.1. Comparison Criteria

The criteria chosen in this paper are selected to give a better definition and a comprehensive overview of the different identity models and to highlight the challenges of the current and future identity system. In the first place, we investigated the seven criteria from Kim Cameron’s article which is a work initiated to give strength to the notion of a web of trust and introduce the user-centric identity metasystem concept [36]. Moreover, we examined the five respect principles from the respect trust framework article that was written by the Respect Network Corporation in order to clarify the privacy, data protection, and trust across the digital trust network [37]. In like manner, we inspected the ten requirements that have been identified by W3C Verifiable claims group [38], and the principles of SSI in Christopher Allen’s article that gives additional perspective on digital identity coupled with the seven myths of SSI introduced by Timothy Ruff [39]. As a result, the seven criteria selected in our work and presented in Table 1 act as a basis for comparing the identity management models.

#### 3.2.2. Discussion

In this subsection, we compare the four identity models according to the seven criteria we defined. Table 2 presents and summarizes the result of this comparison.

Autonomy or self-government is only addressed in the decentralized model as the users in centralized models are always tied to the service providers; while authority or self-control was the main objective of user-centric identity model but still, the user is chained to vendors, hence the self-control is fully addressed in the decentralized model. Availability was the critical issue in the first centralized identity model, for this reason, the concept was addressed in federated and user-centric identity models which provide a medium availability as still any interruption from identity provider will reject users’ request; decentralized model provides a high access model as an individual is the owner of his identity not just a user. Approval or consent is an aspect related to the self-control of the identity; it’s only available in the user-centric and decentralized model as the growth of internet services emerged the concept. Confidentiality was addressed in all models but not all the models provide the same level of confidentiality or minimal disclosure of the data except the user-centric and decentralized model where the user can select what data can be shared. Tenacity is the fact that an identity can be durable enough and used for a lifetime; the concept is only available in the decentralized model as the identity is not related to any identity vendors or governors and no one can delete it except the identity owner. Interoperability was a big issue in the centralized identity, and then across domain concept was introduced in the federated identity model, notably, the concept was integrated into all followed models.

## 4. eHealth Identity Management Players and Use Cases

### 4.1. Healthcare Key Players

The major players in the healthcare industry according to the Institute of Catholic bioethics are the patients, physicians, employers, insurance companies, pharmaceutical firms, and government organization. Ritz et al. introduced the four key players in the eHealth system and explained the relation between the players, and they can be regarded as the categories of healthcare stockholders [40]. In this work we present the four key players as follow:

- Healthcare regulators: including government organizations and healthcare institutions like public health departments and hospitals. In the eHealth system, the regulators aggregate data from the other players in order to develop the eHealth framework and update the care metrics.
- Industry representatives: encompass insurance companies, pharmaceutical firms, and health equipment companies. They operationalize the different elements of the healthcare framework and provide advanced solutions based on a range of data collected from the other players.
- Healthcare providers: can be a physician, a nurse, an ambulance service or any other independent provider. They are responsible for delivering care service and coordinate as care team members. They need access to the facilities and patient information as well as other medical records anytime anywhere to deliver a good quality of service.
- Healthcare consumers: the patients are typically the citizens or people who received treatments and care from the healthcare providers. Patients also may want to access their healthcare records via a personal electronic device or share special information with their care provider.

### 4.2. Key Players’ Roles and Tasks

The roles of healthcare key players’ alter from a scenario to another. The verifiable working group at W3C [41] is developing standards for stating and exchanging claims in an easy and secure way. Up to the present time, they introduced four user roles which are the issuer, inspector, subject, and holder and for each role they listed several tasks. In the case of eHealth identity management, a key player can hold one or more user roles depend on the healthcare scenario as shown in Table 3.

The roles definition of each key player represented in the table are related to the relationships between the stockholders and the viewpoint of different key players to the eHealth assets. As an illustration, a healthcare record database can be regarded as an asset where healthcare regulators can access it for analysis purposes and extract national health metrics, and to an industry representative, it represents reimbursable services or opportunity to develop new drugs and medicines. From the healthcare providers’ point of view is a billing as well as an electronic medical record for his patients, in the same manner, it’s a personal record for the patient and from an insurance standpoint, an audit log of his benefits.

### 4.3. eHealth Identity Scenarios

Healthcare data are confidential data, and the healthcare consumers and organizations seek max security and privacy to protect their information and they are unwilling to distribute their data for non-clinical care purpose, and they like to have full control of giving and revoking data access and to be asked before releasing healthcare information [42]. Table 4 gives several use cases of healthcare identity and identify the participating entities and the requirements.

In all, eHealth identity management system should protect user identity and provide user-related medical data anytime anywhere, since eHealth deals with sensitive data, there is a big need for further security and privacy investigation [43]. While previously we introduced the principles of identity management, the interoperability is a pivotal aspect which refers to the ability of the system to be widely used and adopted by other systems and organization locally and globally. One of the key aspects to make a system widely used is to create a data standard to exchange information seamlessly and reliably. The data standard should be selected carefully answering the applicability, durability, consistency, and sustainability. Anonymity is another important eHealth security feature that answers the sharing of the medical data A to an entrusted receiver B without disclosing identities or tracking the data back to particular individuals. Hence, automate the workflow of particular use cases by reducing manual intervention can provide more security and prevent human failures.

### 4.4. The Limitation of Centralized Identity Management Models for ehealth

The traditional centralized identity management approaches can poorly support the fast-growing of healthcare services and the near horizon of the on-demand digital healthcare ecosystem [44]. The promising healthcare ecosystem will be composed of several stockholders including patients, clinics, professionals, pharmacies, insurance companies and federal government organizations, each stockholder will implement and interpret policies and legal requirements to meet the accountability inside the ecosystem. The eHealth paradigm by nature is decentralized where patient have different gadgets and embedded devices generating medical data anytime anywhere and the patient can visit different clinics and physicians for assistance, while purchasing medicines from different pharmacies and drug suppliers, moreover patient have to share a certain data to insurance companies and federal organization to answer the government laws [45].

The current centralized approaches can improve the mobility and scalability of the system but they showed a poor performance in term of:

- Privacy, security, and usability: the healthcare data is sensitive by nature and it needs a maximum of security against data breaches and privacy disclosure when exchanging the data especially after enabling third parties’ medical services to interact with the system.
- Single point of failure: centralizing all the patients’ credentials and all identity decisions in one IdP leads to a single point of failure that it can be possible by human mistake or a hardware damage as well as a natural disaster.
- Single point of access for malicious users: centralizing all the credentials in one IdP will also make the system weak and give hackers an easy way to scam users unless providing trained security professionals to maintain and manage the security of this point or the system which is considered as another challenge.
- The complexity of the ecosystem: the complexity of securing and managing such a centralized ecosystem is considerable and cost-effective, particularly when building trust between the stakeholders, and may it need a special department with an army of engineers and security professionals to make it possible.

## 5. Decentralized Identity Management Models

### 5.1. Distributed Ledger Technology for eHealth Identity Management

Most of the traditional IdM solutions are centralized based on one single entity to manage the identities; indeed some solutions proposed a distributed approach to handle the limitation of a single entity management especially the federated and the user-centric models. Despite the different models and protocols, the traditional approaches show different limitation namely weak security, privacy, integrity, and confidentiality. To put it differently, the evolution of internet-based services and the fast-growing of internet of things and the smart world concept make the digital identity part of everyday life and in like manner, the non-human entities such as devices, systems, and companies are also playing an important role and it’s necessary to ensure the trust between all entities when exchanging data. However, the traditional method doesn’t support the identity of objects, and the existing healthcare identity management solutions will be the standstill of the future eHealth paradigm where embedded healthcare devices and gadgets will be used daily and assist patients life. An identifier in eHealth identity management should unambiguously distinguishes between all entities and provide a mechanism to make authenticating and sharing data between physicians, healthcare providers and government institutes more secure and private and answer the portability, confidentiality, integrity, and verifiability of the system.

The DLT is the technology that let an individual to store his identity attributes in a secure ledger anytime anywhere in a protected way where no third parties involved and gives the identity owner the ability to access his information and prove a claim without disclosing any other data such as proving that the owner has a valid social security number without revealing it.

### 5.2. Decentralized Identity Models

#### 5.2.1. Self-Sovereign Identity

Self-sovereign identity is a decentralized user-centric model that gives the user full control of his own identity and information data, in other words, the user is the owner and the administrator of his digital identity and it is independent of any service provider. The implementation of SSI requires three concepts, decentralized identifiers, decentralized identifiers documents, and verifiable claims.

Decentralized identifiers (DID) can be obtained by a user at any time for any purpose. A unique identifier to represent an entity such as people, organization, and devices, and is associated with a DID document as a unique identifier. A DID document is an object containing information about the created DID such as creation time, a list of the public keys related to the associated DID, a list of services where the DID can be used and more. The DID document is expected to be immutable, persistent and fully owned and controlled by the DID owner.

**Figure 1:**
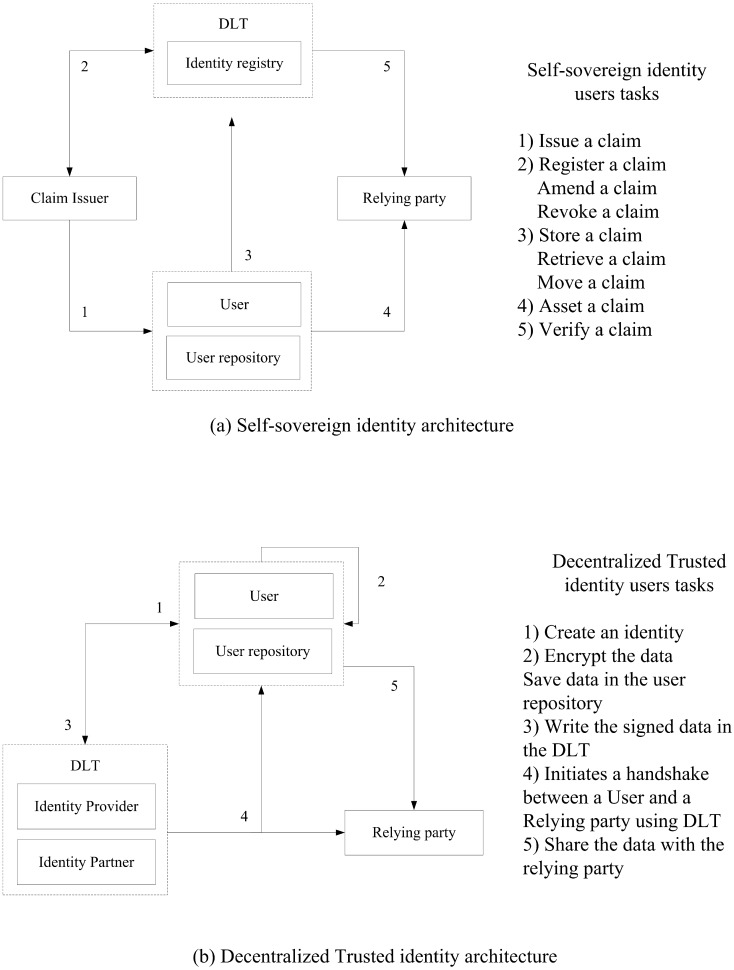
Decentralized identity models.

Variable claims emerged three main actors which are users, claim-issuers, and relying parties. The issuers create claims about the user which are used as attributes of user identity and store them in the identity repository using the DLT. Meanwhile, any relaying parties want to identify a user or verify a claim or a user proof should access the DLT registry after granting permission from the user itself and make an attestation if the claim is true as is shown in Figure 1a [5]. SSI uses three concepts to identify which are claims, proofs, and attestations. A claim is a statement made by a business or a person contains credentials about an entity. Proofs are documents that come in all sorts of formats that evidence a claim like a photocopy of passport or medical bills which are usually stored locally in users’ phones. Attestations are sort of document that is made after a third party validates a claim or proof such as a hospital may attest to the fact that a physician was working there for a period of time.

#### 5.2.2. Decentralized Trusted Identity

In the decentralized trusted identity model, the identity provider still centralized and performs the user’s identity proofing based on trusted credentials like passports or driver licenses while uses the DLT to store the identity attestation for later validation by third parties services and trusted organizations as is shown in Figure 1b [46]. The decentralized trusted identity doesn’t use the concept of identity repository which is the slight difference from the SSI model, and the identity provider will provide the receiving entity a testimony on the validity of the data while all the credentials are encrypted and stored locally in the user phone.

Examples included ShoCard. The decentralized trusted identity was introduced with the growth of the Blockchain technology to make the IdM more secure, efficient and private and fill the gap of the traditional models like usability, privacy, security breaches, and fraud. [47].

## 6. Existing Decentralized Solutions

**Figure 2:**
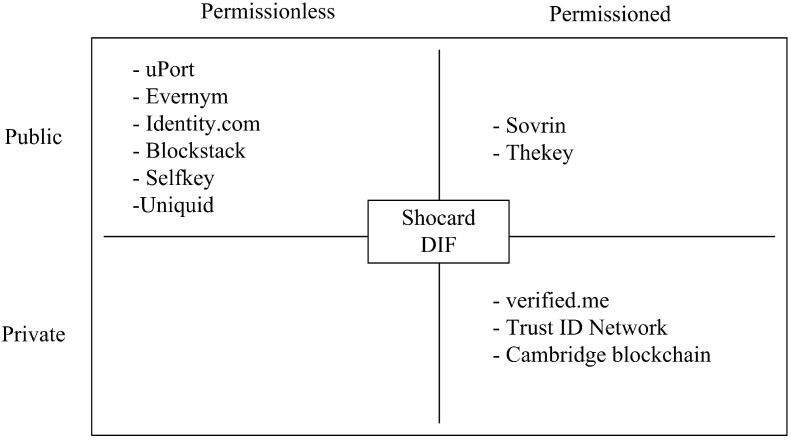
Identity solutions-based distributed ledger technology (DLT) models.

In this section, we will try to give a snapshot of the existing solutions-based DLT that can support eHealth identity management. In the first place, the solutions can be boiled down according to the DLT models. Figure 2 enumerates the different blockchain-based identity management solutions categorized on four metrics public, private, permissioned, and permissionless. As we can see the majority of the existing solutions are made on the top of public blockchains networks like bitcoin and Ethereum and the others are permissioned and build on public and private BC networks while the middle solutions are hybrid, While Table 5 gives a comparison of these solutions.

The Bitcoin is the first peer to peer payment network allows the digital currency Bitcoin (BTC) to be used in a decentralized way by deleting the aspect of central authority and the need of intermediaries while using the cryptographic protocol and the consensus mechanism to achieve a certain level of trust between the peer nodes.

The Ethereum is a public blockchain-based distributed computing platform, it has a native cryptocurrency like Bitcoin called Ether (ETH) but unlike it’s designed to support all kinds of decentralized applications (D-App) by providing a programmable network using solidity scripts which is a high-level language for implementing smart contracts. In this comparison, we excluded the solution that doesn’t have a solid presentation such as published articles, white papers, or wealth documentation.

### 6.1. Public Blockchain Identity Solutions

- uPort is an identity solution settled by ConsenSys Lab [59] and built on the top of Ethereum. The uPort mission is to hand back the control of the digital identity to the user. It uses an interoperable identity layer composed of identity and messaging protocols and it uses also the concept of smart contract and verifiable credentials. The solution infrastructure composed by a mobile app that contains a self-sovereign wallet, an authentication mechanism for the modern web applications and decentralized application, and the developer’s libraries to allow the integration with third party services.
- Sovrin is the solution that claimed to be the decentralized global lifetime public utility for self-sovereign identity [60]. It can be used with any entity including people, organizations, and things. The solution is integrated with Hyperledger Indy which is a blockchain network designed especially for decentralized identity and it’s developed by both Sovrin and Linux foundations.
- Identity.com is an open-source project providing secure access to decentralized identity verification [50]. The ecosystem is composed of Smart contract and blockchain technologies. The solution at first was only designed to support Civic identity system but after that, it becomes a hub for many players as the network grows. The solution has three major components which are smart contracts to leverage the decentralized identity, open-source libraries to allow third-party service to interact with the ecosystem, and open-source application to meet the developer’s needs.
- Evernym is developing a sophisticated identity platform on the top of Sovrin and IOTA [61]. The aim of the Evernym team is to develop the necessary tools and protocols for individuals and enterprises such as identity owner tools, and claim exchange and verification tools to integrate self-severing identity in all industries and ecosystems, and also to leverage SSI identity using different distributed ledger solutions.
- Blockstack is a project built on the top of the Bitcoin network [51] to provide the tools to develop the decentralized application. It’s known as a decentralized identity, discovery and storage platform. The main concept of Blockstack is that computing is performed off-chain and it uses virtual chains to pin the state machines to the network. The identity model in Blockstack known as Onename which is mainly developed to register users and the model is operating on the top of Bitcoin.
- THEKEY is a decentralized ecosystem supporting identity verification using big data and blockchain [52]. THEKEY uses the technology of NEO [62] smart contracts and benefits from the multi-programming language of the NEO network to leverage the identity to various industries. They claim that the second generation of identity verification will be realized by Blockchain-based dynamic multi-dimension identification technology while the project is mainly developed for government purposes.
- SelfKey is an open-source identity system built on the top of Ethereum [53]; consist of three main components which are identity wallet to help the identity owner to access his assets and documents, a marketplace to give user access to various services such as financial, immigration and cryptocurrency, and tokens to power the ecosystem, enable the trust and allow to exchange value.
- Uniquid provides an identity management system for the internet of things and automates access management protocol for the secure machine to machine connections. The solution is to give a device the ability to generate its own identity keypairs using pretty good privacy (PGP) [63] and cryptography principles while making the device able to apply for the needed signature without any centralized authority using blockchain.

### 6.2. Private and Consortium Blockchain Identity Solutions

- Verified.me: SecureKey and IBM are working together to create the first digital identity using blockchain for Canadian leading banks and financial institutions in Canada [56]. The solution will be built on the top of IBM blockchain service which uses the open-source blockchain Hyperledger fabric of Linux foundation. The solution is in testing face.
- Trust ID Network is the platform of Gemalto firm built for decentralized ID management using R3 Corda private blockchain. The aim of the solution is to put users in control of their identities using a mobile app that employs smart contracts, data models and storage mechanisms for increased security. In addition, the solution provides end-to-end data encryption, a zero-knowledge proof protocol for data minimization, and a UX-optimized identity wallet with biometrics for authentication, and ID document verification.
- Cambridge blockchain LLC initiated a solution for financial institutions to meet the strict data privacy rules by eliminating the redundant identity compliance checks and improving the user experience [58]. The solution encompasses four principles which are the user owns his personal data for better control, distributed identity authorities reached by a marketplace for identity services, transparent and secure solution using cryptography and blockchain technologies, and efficiency for all stockholders.

### 6.3. Blockchain Hybrid Identity Solutions

- Shocard is one of the earliest enterprise identity blockchain solutions [55]; it can be run on the top of any blockchain network as it has his own core service that contains blockchain API adapter. It provides multi-factor authentication where a user can log in to apps, devices, and cloud services without a need to a password or a username.
- Decentralized Identity Foundation (DIF) is an engineering-driven organization focused on developing the essential foundational standards to establish an open decentralized identity ecosystem for people, organizations, and devices and ensure the interoperability between all participants. The project is still in the early stage development and it encompasses more than 60 diverse global organizations and contributors working together to establish the ecosystem [64].

### 6.4. Benchmarks for Blockchain-Based Identity Management Systems

To evaluate the different blockchain-based identity management solutions and applications, we discuss the essential metrics of identity management and blockchain benchmarks-based identity management presented in Table 6. Several DLT-based identity management solutions and applications claim the excellent performance and the best evaluation metrics. Still, mostly, the evaluation is based on blockchain performance rather than blockchain-based identity management. In Table 6, we tried to mention some critical aspects of performance metrics for the DLT-based identity management solutions. Yet, the performance metric for such applications needs more work from the research community.

## 7. Challenges and Future Work

The identity management for the promising worldwide on-demand digital healthcare ecosystem is in the first stage of research and implementation. For instance, self-sovereign identity model can be the solution for the eHealth decentralized identity system as it already addressed the major requirements of the decentralized IdM which are governance, performance, accessibility, and privacy using the distributed ledger technology that has the potential to support the integration of the healthcare information across a range of procedures and stakeholders. Despite this, applying the SSI model in healthcare area may give rise to various challenges and limitations including:

- Data standardization: the healthcare organization, government institutes, and insurance companies should consider and give a global format of what identity data should be shared in on or off the distributed ledger and define the size and the format that can be used in the system. A free-form submission of the data to the ledger may be cost-effective and will lead to the creation of unnecessarily large transactions that could harmfully impact the performance of the system. The healthcare policy-makers and different stakeholders should deeply collaborate with companies providing the technology to understand and facilitate the rise of the ecosystem.
- Access permission level: the self-sovereign identity can be implemented on the top of different DLT models, from one hand adopting a public ledger for a global system as it enable broader access may requires proof-of-work calculation which lead to a huge computing power to answer the real-time access for all entities, in the other hand implementing the solution on a private DLT to answer the need of the local healthcare system may requires trust among the participating entities and clear mechanism about reading and writing data to or from the ledger, despite both approaches may have a scalability limitation.
- Scalability limitation: the cost of operating the SSI on a Blockchain technology is not yet known, the famous existing permissionless ledgers like Ethereum and Bitcoin are facing a tradeoff between the transaction volumes and available computing power, for example, Bitcoin can only process around 250,000 transactions daily for around 10 million users, which is not acceptable for the case of real-time identity access. Yet the permissioned ledger can also face a computing power problem due to the poor participation in the network while providing the needed computing power from one side will give it a centralized owner.
- Adoption and incentives for participation: in order to create the promising DLT empower eHealth identity management system, the DLT will need the participation of many individuals to create the network of interconnected computers and provide the needed computing power to process the transaction and maintain the ledger, consequently an incentive approach or a reward system should be designed and tested for healthcare use case and encourage individuals to adopt the proposed approach. Similarly, organizations also should be part of the network and the decentralized system, for this reason, further support is needed to encourage all the stockholders to participate.
- IdM Consensus protocol: the most important element in DLT technology is the consensus protocol, as it provides a controlled and maintained ledger without the need of third parties services. Many consensus protocols are proposed for the case of the cryptocurrencies where for example the order of the event is important to solve the problem of double-spending without an arbitrator. Despite this, identity management may not face the issue of double-spending or an equivalent problem as the IdM paradigm is not transaction-based as currency, for this reason using the existing consensus protocols may not give the performance and the solution needed to support identity management.

## 8. Conclusions

This paper presents the state of the art of decentralized identity management using distributed ledger technology. It discusses the evolution of identity management models and highlights the potential opportunities for adopting the decentralized identity management approaches for future eHealth identity systems. In the first place, we explored the evolution of identity management models, and we conducted a comparison of the four elaborated models regarding the seven principles we adopted in this work, which are autonomy, authority, availability, approval, confidentiality, tenacity, and interoperability. Henceforth, we highlighted the limitation of the centralized identity models toward the eHealth paradigm, and we presented and classified several healthcare identity use cases. Besides, we introduced the self-sovereign identity and decentralized trusted identity models. In the meantime, we revised the existing decentralized identity solutions built on the top of the different distributed ledger technologies, and we discussed several benchmarks to evaluate the exiting solutions. Finally, we elaborate on distinct research challenges and future works.

## Figures and Tables

**Table 1:** The seven criteria of identity management.

Principles	Abstract
Autonomy	Is the individual independent from identity vendors? Individuals must be independent of any identity provider or a central governor.
Authority	Can the individual have full control of his identity? Individuals must have complete power to manage their identities.
Availability	Can an individual have full access to his own data? Individuals must have full permission to gain access to their own data anytime anywhere.
Approval	Can the individual voluntary approve the use of his identity? Individuals must voluntary agree and approve requests before using their identity.
Confidentiality	Can the individual provide his identity with minimal disclosure of data? Individuals should share only the needed credentials with a minimal disclosure
Tenacity	Can the identity live with the individual as long as possible? Individuals identities must be persistent as long as possible
Interoperability	Can the individual identity exchange data with any system or service globally? Individual Identities should be universal and widely used by any entities

**Table 2:** Identity management (IdM) models comparison.

Principles	Centralized Models			Decentralized Models
	Centralized Identity	Federated Identity	User Centric Identity	
Autonomy	No	No	No	Yes
Authority	Low	Low	Medium	High
Availability	Low	Medium	Medium	High
Approval	No	No	Yes	Yes
Confidentiality	Low	Low	Medium	High
Tenacity	No	No	No	Yes
Interoperability	No	Yes	Yes	Yes

**Table 3:** Key players’ roles and tasks.

Key Players	User Roles	Role Definition
Healthcare regulators	Issuer	Regulators can create claims about the authorized clinics, hospitals, and care providers.
	Inspector	Regulators can verify the care providers’ certificates and patients’ assurance validity.
	Holder	Regulators can control and hold different claim about the other key players.
Industry representatives	Issuer	They can create claims about their own products and services to care providers or consumers.
	Inspector	They can verify claims coming from healthcare providers and consumers about particular products.
	Holder	They can control and hold claims about operations and product selling records.
Healthcare providers	Issuer	Care providers can create claims about healthcare consumers and it can be stored as healthcare records.
	Inspector	Care providers can inspect and verify claims of their own patients and industry representative.
	Holder	Care providers can control and hold claims like working certificate.
Healthcare consumers	Issuer	Consumers can create claims about their own health condition using devices and gadgets.
	Inspector	Consumers can verify claims coming from care provider for example in a case of telemedicine and remote care.
	Holder	Consumers can control and hold claims such as medical records.

The subject role is not presented in the table as we consider the holder is the subject. There are few use cases where the subject can be different from the holder, for example a kid can be the subject of the claim but the holder of the claim should be his parents. A holder is typically the controller of the claims.

**Table 4:** The healthcare identity scenarios.

Name	Scenario	Key Players	Requirements
Healthcare data exchange	Visiting a different health clinic in a different stat after feeling sick when traveling for a vacation or business. The patient will aim to provide only the requested and required information that verifies his identity by delivering an identity credential without disclosing other information like social security number or marital status, besides he wants to revoke the access to his information after a set of time	-Consumers -Regulators	Interoperability is the main aspect to exchange data between different healthcare institutions in different states and countries.
Online pharmacy	An online pharmacy may receive a patient prescription from a local clinic. The pharmacy needs to verify the identity of the physician and his ability to write a prescription, as well as the identity of the patient and his insurance coverage before picking up his medication	-Consumers -Care providers -Industry representatives	*Automate* the essential cases and prevent a manual intervention for better security while providing strong access, authentication, and authorization mechanisms.
Payment	A company started an employee assistance program with the aim of providing health insurance and treatment coverage to all employees where the company has branches. This health plan requires access to the employees’ medical records who received a treatment, which will be delivered from the healthcare providers in different locations in order to calculate the budget of the services provided.	-Consumers -Industry representatives -Care providers	Data standard is mandatory for exchanging the information between the key players.
Research project	A research project on children is being conducted in a double-blind study and needs access to new data sets for children younger than the age of 13. After authorizing the access to the data, the IRB will review the project and apply research protocols at the medical center where the investigation is located. The research lab asked the principals if they can have access to the data for another 6 months and use the data collected for another study purpose which is not related to the research protocol reviewed in the beginning.	-Consumers -Industry representatives -Regulators	Anonymity is the key feature to share the data of a group of consumers without disclosing their identity.

**Table 5:** Decentralized identity management solutions.

Identity Project	DLT Model	DLT Platform	Identity Model	Launch Date	Open Source	Consensus Algorithm	Industry Target
uPort [48]	Public Permissionless	Ethereum	Self-sovereign	January 2017	Yes	PoW (Ethash)	-
Sovrin [49]	Public Permissioned	Hyperledger Indy	Self-sovereign	July 2017	Yes	Redundant Byzantine Fault Tolerance (RBFT)	-
Identity.com [50]	Public Permissionless	Ethereum	Decentralized Trusted Identity	Later	Yes	PoW (Ethash)	-
Evernym [49]	Public Permissionless	IOTA	Self-sovereign	Later	Yes	Consensus achieved by Confirmation confidence	Finance, Education, Healthcare, Commerce, Travel
Blockstack [51]	Public Permissionless	Bitcoin	Self-sovereign	2013	Yes	PoW (Proof-of-Work)	Software
Thekey [52]	Public Permissioned	NEO	-	November 2014	No	Delegated Byzantine Fault Tolerance (dBFT)	Software
Selfkey [53]	Public Permissionless	Ethereum	Self-sovereign	2016	Yes	PoW (Ethash)	Financial services
Uniquid [54]	Public Permissionless	Bitcoin	-	October 2015	Yes	PoW (Ethash)	IoT
ShoCard [55]	Can support all DLT models	-	Decentralized Trusted Identity	May 2015	No	Not specified	Enterprises, financial institutions, airlines, website and app login
Sovrin [49]	Public Permissioned	Hyperledger Indy	Self-sovereign	July 2017	Yes	Redundant Byzantine Fault Tolerance (RBFT)	-
verified.me [56]	Private Permissioned	Hyperledger Fabric	Self-sovereign	Late 2017	No	Byzantine fault tolerance (BFT)	Banking
Trust ID Network	Private Permissioned	R3 Corda	Self-sovereign	September 2018	No	Consensus over state validity and uniqueness	Banking and financial institutions
DIF [57]	Can support all DLT models	-	Self-sovereign	Later	Yes	Not specified	All industries
Cambridge blockchain LLC [58]	Private Permissioned	-	Decentralized Trusted Identity	2015	No	-	Financial institutions

**Table 6:** Blockchain-based identity management systems metrics.

Metrics	Explanation
Time spent provisioning and de-provisioning users	Provision and de-provisioning is the core functionality of any identity systems, managing the user accounts from registration to authentication and updates in a short time is a mandatory metric for mature identity management systems. Delays of those operations or failed Logins may lead to a failure of the solution [65].
Unique accounts per user	The more s user creates several accounts the more he is to forget about the credentials. A user may create more than one account for different purpose especially when the process of recovering the username or the password is hard. Single Sign-On (SSO) platforms are mainly to solve this problem [65].
Password reset volume per month	Many companies and applications require changing their password periodically to ensure strong security systems. A mature IdM should answer to the requirement and makes it easy for users to change their password is a fast and secure ways [66].
Number of uncorrelated accounts	Many accounts may not have owners due to changing accounts, upgrading the system, or a user may leave his account open without using it. This situation may lead to a security threat. A mature system my monitor such accounts to reduce the unnecessary risks [66].
Read latency	Reading from the ledger is the most performed action in blockchain networks which is the time between sending the reading request until the replay is received. Improving the reading latency will help to optimize the authentication time [67].
Read throughput	The metric of defining how many reading operations are performed in a period of time. This metric is not really relevant for blockchain network but important for blockchain-based identity solution as most solutions relay to blockchain performance, not to the identity system needed [67].
Transaction latency	Blockchain transactions are time-consuming as the consensus process is used to make the transaction valid across the network. Transaction with long delays may cause a system failure as is not providing a good user experience [68].
Transaction throughput	It’s the rate of valid transaction committed in a set of time. Mainly this metric is performed in every node of the blockchain network to have accurate results and to identify the issues if any. In the blockchain-based identity system transaction throughput is connected to provisioning and de-provisioning operations [68].
Actual production usage	Knowing the real system usage will help to specify the metrics. For instance, in the identity management systems, provisioning and authentication are the most performed operations that it matches in the blockchain the reading and the transaction requests. The more we understand the system the more we easily and accurately identify the metrics and perform evaluations [69].
